# APP: an Automated Proteomics Pipeline for the analysis of mass spectrometry data based on multiple open access tools

**DOI:** 10.1186/s12859-014-0441-8

**Published:** 2014-12-30

**Authors:** Erik K Malm, Vaibhav Srivastava, Gustav Sundqvist, Vincent Bulone

**Affiliations:** Division of Glycoscience, School of Biotechnology, Royal Institute of Technology (KTH), AlbaNova University Centre, Stockholm, Sweden

**Keywords:** Proteomics, Automation, Validation, Distributed processing

## Abstract

**Background:**

Mass spectrometry analyses of complex protein samples yield large amounts of data and specific expertise is needed for data analysis, in addition to a dedicated computer infrastructure. Furthermore, the identification of proteins and their specific properties require the use of multiple independent bioinformatics tools and several database search algorithms to process the same datasets. In order to facilitate and increase the speed of data analysis, there is a need for an integrated platform that would allow a comprehensive profiling of thousands of peptides and proteins in a single process through the simultaneous exploitation of multiple complementary algorithms.

**Results:**

We have established a new proteomics pipeline designated as APP that fulfills these objectives using a complete series of tools freely available from open sources. APP automates the processing of proteomics tasks such as peptide identification, validation and quantitation from LC-MS/MS data and allows easy integration of many separate proteomics tools. Distributed processing is at the core of APP, allowing the processing of very large datasets using any combination of Windows/Linux physical or virtual computing resources.

**Conclusions:**

APP provides distributed computing nodes that are simple to set up, greatly relieving the need for separate IT competence when handling large datasets. The modular nature of APP allows complex workflows to be managed and distributed, speeding up throughput and setup. Additionally, APP logs execution information on all executed tasks and generated results, simplifying information management and validation.

**Electronic supplementary material:**

The online version of this article (doi:10.1186/s12859-014-0441-8) contains supplementary material, which is available to authorized users.

## Background

Tandem mass spectrometry (MS/MS) analyses of heterogeneous protein samples typically provide large amounts of data. Current methods allow the identification of hundreds or even thousands of proteins from a given complex sample. But the acquisition of such comprehensive MS data may require long machine running times, sometimes measured in weeks, depending on the type of analysis and number of replicates performed. The thorough analysis of the complex data produced can be achieved using multiple tandem MS search engines and other specialist tools. However, the handling of proteomics data is not a trivial task. In addition, processing can be very demanding in terms of computer resource and laboratories without a specialized computer infrastructure are limited in their analytical capacity. In order to address these issues, we have developed an automated proteomics pipeline designated as APP that integrates a set of tools and allows distributed execution on most available computers with minimal setup. APP also addresses usability issues and minimizes the computer skills needed to perform complex analyses. It is readily usable in any laboratory environment, both locally and with regards to cloud computing.

## Implementation

**Figure 1:**
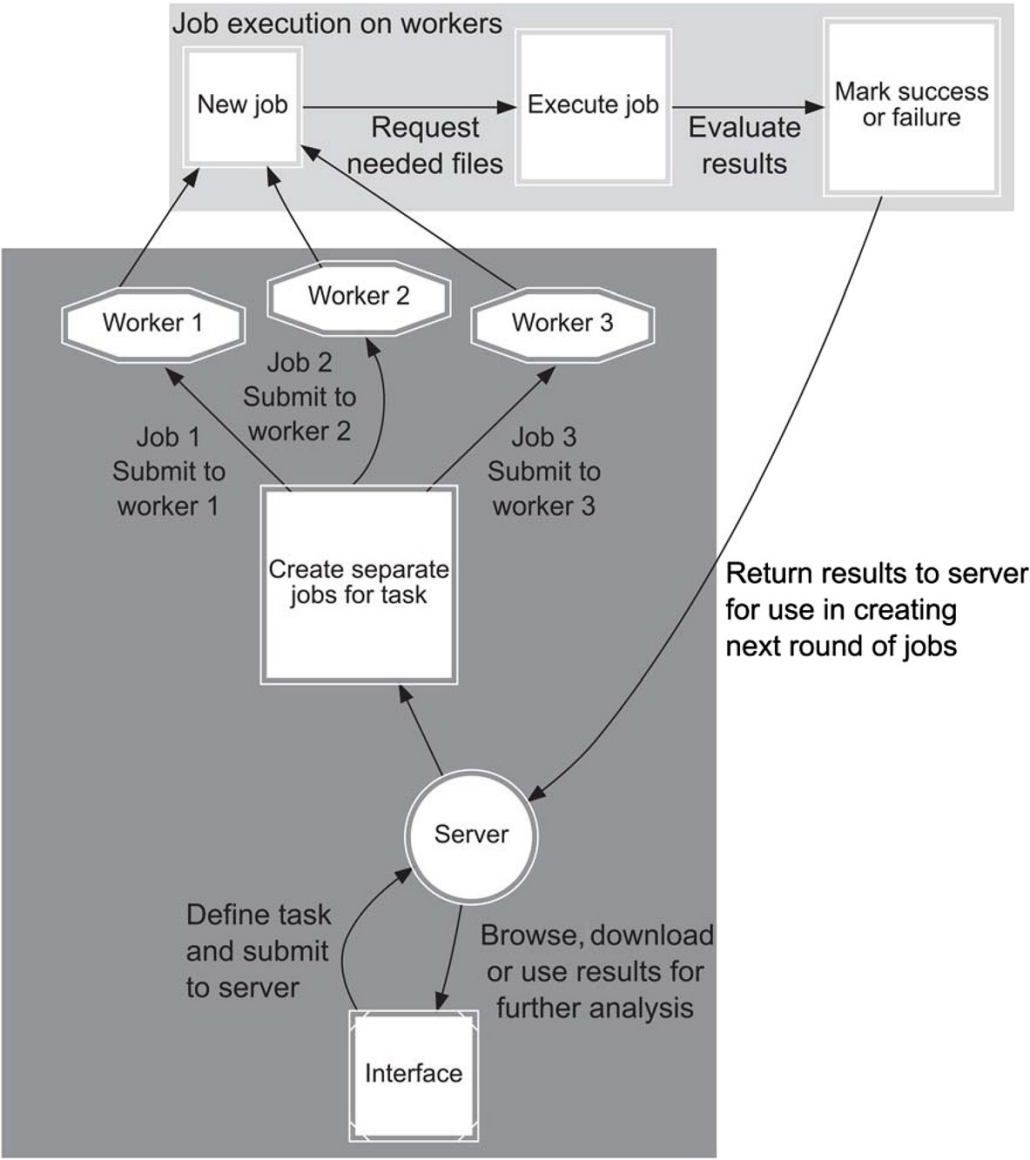
**Job handling on APP.** Overview of the general APP server and worker setup with jobs being distributed and executed on workers.

The core components and plugins of APP were written in Java version 7 using the Sun Netbeans IDE environment (http://netbeans.org). Java was selected to aid deployment over heterogeneous computer environments, thereby allowing Windows, OS X and Linux machines to provide their own unique tools. APP is used through two applications, *i.e.* a server/worker application and a user interface application (Figure 1). The APP project homepage provides prepackaged bundles of all tools and a tutorial manual with step-by-step instructions for server setup, installation of all components and use of APP for the analysis of MS data. The bundles are regularly updated to coincide with TPP releases. A processing node functioning on either a Linux or Windows system becomes available after unpacking the single zip/tar.gz file corresponding to the packaged bundles. Any adapted settings of APP can be mirrored by simply copying the corresponding folder into a new system. APP grids are built to function with minimal privilege. Only the server needs to be accessible to the clients or nodes, thereby placing no requirement for administrative privilege on the other systems. APP does not aim to supplement systems management tools for large deployments. If resources such as high-performance shared storage are available, APP can utilize them but this is not a requirement. APP can utilize binaries from any location, including network shares, thereby allowing access of shared folders to all APP nodes, or folder syncing. APP allows file download from the server to local drives for further work on the files. File security on the nodes falls outside the reach of APP and the software does not add any demand of accessibility to nodes.

### Plugin model

Proteomics and other tools are provided by APP as plugins. Each plugin has its own first in/first out (FIFO) type of queue. Individual plugins use a set of files as input and process them before specifying which output files should be passed on to the next plugin. Thus, complex tasks can be defined easily by linking sets of plugins. Most of the plugins depend on open source tools, allowing the backend tools to be distributed along with the APP server software.

### Server software

The server software handles the central parts of APP, job submission, execution scheduling, timing out non-responsive tasks and tracking of results. It can also be run in worker mode, allowing it to connect to an already running server, and be served single jobs to execute, acting as a simple grid node (Figure 1). This offloads computing tasks from the main server and allows parallel processing of jobs without requiring the complex task of setting up a standard grid or rewriting any software for distributed computing. Workers can be added and removed during runtime, allowing use of computers as they become available. For example, office computers can be used to process long running tasks overnight and then be reclaimed by their owners without affecting the overall processing. Virtualized servers can also be added easily to cope with increased demand. This allows easy use of local computers or computing resources from a cloud computing service such as Amazon Web Services or Microsoft Azure. Since the APP core is written in Java, the server software is inherently cross-platform. Most plugin codes are automated to find and use either Linux or Windows binaries, thereby enabling easy mixing of GNU/Linux and Windows systems. Plugins for use with the Trans-Proteomic Pipeline (TPP) are included with all installations of APP along with a set of freely redistributable database search engines and their plugins. Each worker client can be configured independently. Possible setups include a central server with a set of desktop computers connected for executing tasks at low priority, allowing computer power not needed for desktop tasks to be used by APP. In other setups, specific search engines are executed only on computers with enough computing power while slower computers handle less computationally intensive tasks. APP can accept data in different formats at any stage of the analysis, including raw MS data from vendors such as Waters, Agilent or Thermo Scientific, as well as mgf, pkl and dta files. APP automatically manages file formats containing fixed paths. All APP jobs are executed using a temporary path structure to ensure non-destructive processing and the jobs are updated to their primary paths upon successful completion. Thus, execution can indifferently take place locally or remotely. Files generated on the server can be referenced directly when submitting new jobs, avoiding needless transfer and replication of data. Nodes have tasks submitted and then request needed files. Two methods are deployed to avoid bottlenecks. By default, each node accepts 110% worth of its free core in jobs. This allows file transfers to be performed while the node is processing important jobs. Additionally, file checksums are stored when received and sent. Thus, files that are repeatedly used, such as databases, are only transferred once to the node. The file cache is cleared after no job has utilized a file for a preset time, with the smallest files being cleared first. Finally, APP can be pointed to folders containing commonly used files, which will then be passed by reference rather than transferred. This is useful for storage of databases on all nodes or mapping of shared storage solutions. Very large files such as .raw files can cause bottlenecks for initial transfer to processing nodes, but this is normally not rate limiting because processed raw data are transferred faster than they are processed.

**Figure 2:**
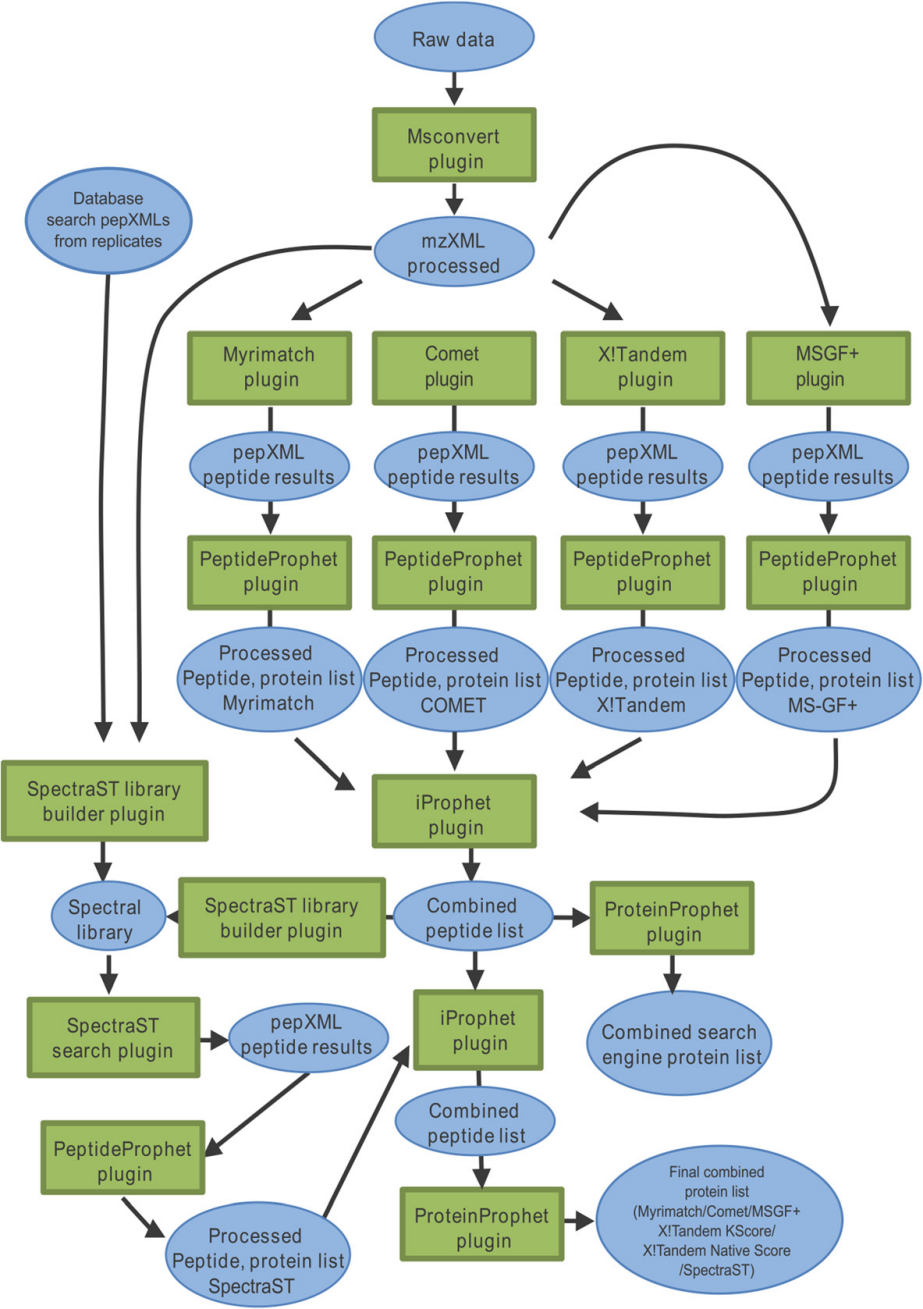
**Sample task.** All workflows on APP clusters are provided as a set of linked plugins. The figure shows how the major plugins are linked in the example task. Housekeeping plugins such as SpectrumNamefixer and IDConvert are excluded for legibility.

The most salient advantage of APP is the integration of multiple plugins for a whole range of tools (Figure 2). This is illustrated by the combination of currently available plugins, *i.e.* MsConvert (ProteoWizard) for raw data format conversion, and database search engines such as X!Tandem [1], Myrimatch [2], OMSSA [3], Comet [4], InsPecT [5], SpectraST [6] and MS-GF+ [7]. The current pipeline also integrates several plugins for validation of the search results, namely PeptideProphet [8], including support for LIBRA, XPRESS and ASAPRatio for iTRAQ or SILAC quantitation [9], iProphet [10] and ProteinProphet [11]. In addition, a separate general search settings plugin reads post-translational modifications in Unimod format [12] and generates a universal parameter format that can be used for all search engine plugins. A label-free quantitation plugin is also available, which can utilize spectral counting or MS2 TIC values [13] as a basis of protein quantitation. Additional plugins provide smaller services such as standardizing the names of spectra, allowing extraction of specific peptide information and images of spectra, or converting additional formats such as BioLynx XML files into appropriate formats for use in APP workflows. APP is packaged together with most of the tools it supports, such as the TPP base installation and all database search engines supported. In cases where underlying software cannot be legally redistributed, plugins can be downloaded individually from the project homepage. The server software typically runs with a user interface to allow easy configuration, but it can also be run completely from a command-line interface for use on server operating systems. It is also possible to configure a server using the GUI and then run it headless.

### Interface software

The interface application is designed to be as intuitive as possible, automatically detecting any APP servers running on a local network and facilitating the setup and submission of complex pipelines by simply linking one plugin to another. The interface also allows users to browse previous tasks and results from the server, easily retrieving any file for local storage. When setting up a task APP pre-calculates the result of each operation, allowing browsing of exactly which output files will be created and forwarded in the task. When attempting to submit a task for processing, a series of checks are run to ensure that each plugin will be provided with proper inputs, for example ensuring that a database search engine is receiving a search-settings file, at least one data file and a target database. Any plugin that fails a test is highlighted to the user and its requirements are presented.

## Results and discussion

### APP and similar software

Several commercial platforms for the analysis of MS-based proteomics data, such as Sorcerer (Sage-N Research), SCAFFOLD (Proteome Software) and PEAKS (Bioinformatics Solutions Inc.) are available, but their access is typically expensive. Other pipelines that incorporate solutions for analyzing and validating results from several MS/MS search engines are freely available, *e.g.* OpenMS/TOPP [14], TPP [15], CPAS (Labkey Software Foundation) and MASPECTRAS [16]. However, they typically require specific knowledge of each supported search engine, such as parameter formats and supported input and output formats. Compared to the abovementioned tools, APP has the advantage of offering a user-friendly proteomics server software enabling distributed computing with minimal configuration. The flexible plugin model allows the establishment of unique proteomics pipelines tailored to the needs of the data. This makes routine use of complex workflows feasible and allows simple scaling of processing capacity with demand. Since submitted tasks can be continuously monitored during execution, APP provides feedback at every step of the process and is never a black box for the user.

The need for a general solution to distribute software tools has been acknowledged by projects such as Taverna Workbench (http://www.taverna.org.uk/) [17], YABI (http://www.ncbi.nlm.nih.gov/pmc/articles/PMC3298538/), Knime (http://tech.knime.org/community/bioinf/openms) and Galaxy (https://usegalaxyp.org/). However, these depend on the maintenance of one or several grid engines to provide distributed and remote processing. Knime relies on a functional setup of Sun GridEngine. Likewise both Galaxy and Taverna Workbench depend on the establishment and maintenance of an existing computational cluster. YABI can utilize a number of such grid engines, but does not fundamentally change the need for advanced setup and maintenance. This is beyond both the abilities and needs of many research groups. APP focuses on simplifying setup of IT infrastructure on commodity computers and does not depend on any tool outside of the core application for distributed processing. Any virtual or physical machine capable of executing APP can easily be made part of its processing network. For this reason, the task of setting up and maintaining a processing infrastructure is practical for “pure research” groups and precludes the need for access to core shared infrastructure.

APP aims to make task execution as simple and powerful as possible by providing distributed processing for multiple search engines compatible with TPP. For advanced and automated workflows, it is possible to script pipelines, but this places great demands on user computer skills and does not by default handle distribution, parameter generation or job monitoring. Unlike the Petunia web-based interface provided by TPP, APP addresses these issues by providing a number of template tasks and default optimized executions without any need to know specific commands or parameter input formats. The APP plugin model preserves much of the flexibility of the scripting approach. Thus, APP combines the great flexibility of scripted pipelines while providing a simple way to execute standard tasks. Users are encouraged to refer to the tutorial section of the APP manual available on the project homepage to gain a good understanding of the APP task model.

### Example of a workflow

An example of data processing using APP is presented in Figure 2. The data representing an in-gel plasma-membrane digest of the recent proteomics study of Srivastava et al. [18] were used for the analysis. They were converted from 48 mgf files into 144 mzML files using the MSConvert plugin, each mzML file containing a portion of the spectra from its parent file. This was done to allow more efficient parallelization of data processing. The task included multiple database search engines, *i.e.* Comet, MS-GF+, Myrimatch and X!Tandem using both K!Score and Native scores. Searches were performed against the black cottonwood (*Populus trichocarpa*) protein sequence database [19] concatenated to its own reverse protein sequences. Searches were performed using tryptic settings, with one tryptic missed cleavage allowed. A precursor tolerance of 50 ppm and a fragment tolerance of 0.1 Da were used. The fixed peptide modification selected was ethanoylated Cys. A single variable mode for oxidized Met was used. The Myrimatch output was processed through the APP SpectrumNameFixer plugin to standardize the naming of the spectra. MS-GF+ had its output mzID files converted to pep.xml using IDConvert, prior to the standardization of the names of the spectra with the SpectrumNameFixer plugin. Results from each search engine were validated using PeptideProphet/iProphet and ProteinProphet. The iProphet output from each search engine was then combined using iProphet and a resultant protein list created using ProteinProphet. A spectral library was then constructed using high scoring peptide spectral matches (PSMs) from the combined iProphet result. This spectral library was combined with search results from the two additional gel samples ran previously in [18]. Decoy spectra were added to the final spectral library at a ratio of 1 decoy spectrum per real spectrum. SpectraST was then used to perform a spectral search on all files utilizing the newly generated spectral library. The spectral search results were analyzed using PeptideProphet/iProphet and database and spectral search outputs were combined using an iProphet plugin. A ProteinProphet plugin was used to create the final protein list. The input data contained around 58000 MS/MS spectra. Two setups were used to compare results from centralized and distributed executions: (*i*) a single high-end compute optimized Amazon instance was used to process the task, which is equivalent to the utilization of a single very powerful computer; (*ii*) several smaller compute optimized instances were used to illustrate distributed processing over several smaller nodes.

Amazon computing power can be measured in virtual CPUs (vCPU). The total number of vCPUs was kept constant, with 32 vCPUs for the single c3.8xlarge compute instance and an equivalent 32 vCPUs for the 8 c3.xlarge Amazon instances (distributed execution). This approach illustrates any impact of APP scheduling on task speed. While the network IO and drives are faster for the single powerful instance, this difference is less important than pure processing power since APP handles file transfers during processing of previous jobs, allowing workers to process continuously while jobs are provided. A second task was also submitted to the APP server. In this case, the Label Free Data Extractor plugin was used to export peptides and PSMs from all combined results and search engines, using the following criterion: each ProteinProphet protein with a probability above 0.95 had supported peptide sequences with a probability above 0.9 indexed. These had all post-translational modifications (PTMs) stripped and each supporting PSM with a probability above 0.5 was exported. PSMs with a probability above 0.5 are treated as supportive by ProteinProphet and thus gave a positive contribution to peptide probability. The exported results were filtered to keep only a single instance of any repeat PSMs or stripped peptide sequences. Additionally, before counting unique peptide sequences all Leu residues were replaced with Ile since these residues are indistinguishable by MS.

### Results from the workflow used as an example

The results obtained from the above example workflow are depicted in Additional file 1: Figures S1 and S2, and summarized in Table 1. The versions of the software used are listed in Additional file 1: Table S1. The execution time for all tasks in the distributed setup was 6 h whereas the single instance required a somewhat longer execution time of about 8 h. It is noteworthy that a majority of computation time was spent to process the MS-GF+ search results, with an average processing time of over 1 hour per data file in both setups (Table 1). This is more than at least 10 times longer than needed for the other search engines to process the same data. It is thus a limitation for users with limited computer resources. However, MS-GF+ search jobs are run in parallel, which uses less total time than sequential runs of jobs. Some differences in speed were experienced with different plugins running on a single instance compared to the distributed execution. Typically, Comet was slightly faster in the single instance execution mode, while X!Tandem with Native score was slower. Likewise, Myrimatch processed its results slightly faster using a single node, while MS-GF+ was slower despite the fact that the amount of memory available and disk speed is advantageous in the single node execution setup. Most differences in processing speed are offset by increased parallelism in the distributed task, with Myrimatch executing 8 parallel instances when distributed, but only a single job utilizing all 32 vCPUs on average when running on the single instance mode. Overall, the observed execution times show that distribution of processing is at least as performant for large tasks as runs on a single powerful computer. This illustrates the ability to assemble an efficient processing infrastructure from multiple slower nodes.

**Table 1:** **Summary of number of hits and processing time for each search engine (seconds to execute a search job using 1000 MS/MS spectra; note that many such jobs can be run in parallel)**

	**X!Tandem: Native score**	**X!Tandem: K-score**	**Myrimatch**
PSMs	3457	6166	6348
Peptides	1594	2357	2337
Average execution time (s) [distributed]	25	26	345
Average execution time (s) [single instance]	26	26	375
	**MS-GF+**	**SpectraST**	**Comet**
PSMs	8655	1050	6155
Peptides	3407	578	2312
Average execution time - Distributed	3747	18	30
Average execution time - Single instance	4477	20	14
	**All database search**	**All DB Search and SpectraST**	
PSMs	13029	13232	
Peptides	3505	3501	

See Additional file 1: Figure S1 for an overview of the output provided by each search engine.

### Future developments

APP is a plugin-based infrastructure with virtually unlimited opportunities for expansion. It allows the management of all aspects of proteomics analysis through a single interface. APP distributes the processes and wraps applications in a way that does not require their re-writing. It will be continuously upgraded through the implementation and addition of new plugins. For example, we are currently integrating an external MASCOT server support (http://www.matrixscience.com) as well as support for MSBlender [20] and IDPicker [21] to allow other validation pathways. More integration targets are UniNovo [22] for use in *de novo* sequencing and Blast2GO [23] for the functional annotation of search results, along with plugins for the generation of inclusion and exclusion lists for use in repeated MS/MS runs. Plugins allowing the structured export of data from APP projects into other data backends will also be released to aid integration of APP with an external database repository or other analysis tools. The additional plugins will be released on the project homepage as they are tested and pass quality controls.

## Conclusions

APP is a user-friendly and powerful tool that allows research groups to easily set up and perform distributed proteomics processing. Its modular plugin nature greatly facilitates complicated proteomics tasks, such as analysis by several database search engines. APP also greatly simplifies information management and provides multiple tools for non-search related tasks, including spectral counting or housekeeping tasks such as fixing spectral references in search engine outputs. Deploying APP computing nodes for database searches is as easy as unzipping a single archive. Thus, even groups without dedicated IT support are able to perform large scale distributed computing independently. The parallel utilization of several search engines provides significant advantages over the use of a single engine, both in terms of coverage and validation. Each search engine identifies a number of unique peptide sequences and spectra that are missed by other search engines. Individual engines also provide their own scores for use in validation of commonly identified peptides. In the example provided here, MS-GF+ and Myrimatch identified the largest number of spectra and peptides, whereas Myrimatch and X!Tandem with K-Score identified the largest number of peptide sequences not matched by another search engine. Comet and X!Tandem have a very high performance and might be the recommended pair of search engines to use when performing searches on limited hardware.

## Availability and requirements

**Project name:** Automated Proteomics Pipeline.

**Project home page:**https://sourceforge.net/projects/automatedproteo.

**Programming language:** Java version 7.

**Operating system:** Platform independent, packaged for Windows and Ubuntu.

**Other requirements:** Java runtime 1.7 or higher; the web portion requires Perl 5.16 and Apache web server.

## Additional file

Additional file 1:
**Table S1.** Software versions used for example tasks. **Figure S1.** Output of the example workflow. a) Number of identified peptide sequences by each search engine; while many peptides are common, each search engine provides a unique set of identified peptides. b) Equivalent output as in a), but for PSMs; the figure highlights matched spectra rather than peptide sequences. **Figure S2.** Output of the combined workflow. Venn diagrams showing the extent of overlap between different combinations of search engines. a) and b) represent overlap of unique identified peptide sequences and PSMs, respectively. Note that iProphet filters out low-scoring PSMs and peptides.

## Abbreviations

APP: Automated proteomics pipeline
TPP: Trans proteomic pipeline
MS/MS: Tandem mass spectrometry
PSM: Peptide spectral match
PTM: Post-translational modification
vCPU: Virtual central processing unit

## References

[1] Craig R, Beavis RC (2004). TANDEM: matching proteins with tandem mass spectra. Bioinformatics.

[2] Tabb D (2007). MyriMatch: highly accurate tandem mass spectral peptide identification by multivariate hypergeometric analysis. J Proteome Res.

[3] Geer LY, Markey SP, Kowalak JA, Wagner L, Xu M, Maynard DM, Yang X, Shi W, Bryant SH (2004). Open mass spectrometry search algorithm. J Proteome Res.

[4] Eng JK, Jahan TA, Hoopmann MR (2013). Comet: an open-source MS/MS sequence database search tool. Proteomics.

[5] Tanner S, Shu H, Frank A, Wang L, Zandi E, Mumby M, Pevzner P, Bafna V (2005). InsPecT: identification of post-translationally modified peptides from tandem mass spectra. Anal Chem.

[6] Lam H, Deutsch E, Eddes J, Eng J, King N, Stein S, Aebersold R (2007). Development and validation of a spectral library searching method for peptide identification from MS/MS. Proteomics.

[7] Kim S, Mischerikow N, Bandeira N, Navarro JD, Wich L, Mohammed S, Heck AJR, Pevzner PA (2010). The generating function of CID, ETD, and CID/ETD pairs of tandem mass spectra: applications to database search. Mol Cell Proteom.

[8] Keller A, Nesvizhskii A, Kolker E, Aebersold R (2002). Empirical statistical model to estimate the accuracy of peptide identifications made by MS/MS and database search. Anal Chem.

[9] Li X-J, Zhang H, Ranish JA, Aebersold R (2003). Automated statistical analysis of protein abundance ratios from data generated by stable-isotope dilution and tandem mass spectrometry. Anal Chem.

[10] Shteynberg D, Deutsch EW, Lam H, Eng JK, Sun Z, Tasman N, Mendoza L, Moritz RL, Aebersold R, Nesvizhskii AI (2011). iProphet: multi-level integrative analysis of shotgun proteomic data improves peptide and protein identification rates and error estimates. Mol Cell Proteom.

[11] Nesvizhskii A, Keller A, Kolker E, Aebersold R (2003). A statistical model for identifying proteins by tandem mass spectrometry. Anal Chem.

[12] Creasy DM, Cottrell JS (2004). Unimod: protein modifications for mass spectrometry. Proteomics.

[13] Asara J, Christofk H, Freimark L, Cantley L (2008). A label-free quantification method by MS/MS TIC compared to SILAC and spectral counting in a proteomics screen. Proteomics.

[14] Kohlbacher O, Reinert K, Gröpl C, Lange E, Pfeifer N, Schulz-Trieglaff O, Sturm M (2007). TOPP–the OpenMS proteomics pipeline. Bioinformatics.

[15] Keller A, Eng J, Zhang N, Li XJ, Aebersold R (2005). A uniform proteomics MS/MS analysis platform utilizing open XML file formats. Mol Sys Biol.

[16] Hartler J, Thallinger GG, Stocker G, Sturn A, Burkard TR, Körner E, Rader R, Schmidt A, Mechtler K, Trajanoski Z (2007). MASPECTRAS: a platform for management and analysis of proteomics LC-MS/MS data. BMC Bioinformatics.

[17] de Bruin JS, Deelder AM, Palmblad M (2012). Scientific workflow management in proteomics. Mol Cell Proteom.

[18] Srivastava V, Malm E, Sundqvist G, Bulone V (2013). Quantitative proteomics reveals that plasma membrane microdomains from poplar cell suspension cultures are enriched in markers of signal transduction, molecular transport and callose biosynthesis. Mol Cell Proteom.

[19] Tuskan GA, Difazio S, Jansson S, Bohlmann J, Grigoriev I, Hellsten U, Putnam N, Ralph S, Rombauts S, Salamov A, Schein J, Sterck L, Aerts A, Bhalerao RR, Bhalerao RP, Blaudez D, Boerjan W, Brun A, Brunner A, Busov V, Campbell M, Carlson J, Chalot M, Chapman J, Chen G-L, Cooper D, Coutinho PM, Couturier J, Covert S, Cronk Q (2006). The genome of black cottonwood, Populus trichocarpa (Torr. and Gray). Science.

[20] Kwon T, Choi H, Vogel C (2011). MSblender: a probabilistic approach for integrating peptide identifications from multiple database search engines. J Proteome Res.

[21] Ma ZQ, Dasari S, Chambers MC, Litton MD, Sobecki SM, Zimmerman LJ, Halvey PJ, Schilling B, Drake PM, Gibson BW, Tabb DL (2009). **IDPicker 2.0: improved protein assembly with high discrimination peptide identification filtering.***J*. Proteome Res.

[22] Jeong K, Kim S, Pevzner PA (2013). UniNovo: a universal tool for *de novo* peptide sequencing. Bioinformatics.

[23] Conesa A, Götz S, García-Gómez JM, Terol J, Talón M, Robles M (2005). Blast2GO: a universal tool for annotation, visualization and analysis in functional genomics research. Bioinformatics.

