# Supplementary material for: APP: an Automated Proteomics Pipeline for the analysis of mass spectrometry data based on multiple open access tools
Source: BMC Bioinformatics. 2014 Dec 30;15(1):441. doi: 10.1186/s12859-014-0441-8 (PMC4314934; doi:10.1186/s12859-014-0441-8)
Supplement: Additional file 1: — Table S1. Software versions used for example tasks. Figure S1. Output of the example workflow. a) Number of identified peptide sequences by each search engine; while many peptides are common, each search engine provides a unique set of identified peptides. b) Equivalent output as in a), but for PSMs; the figure highlights matched spectra rather than peptide sequences. Figure S2. Output of the combined workflow. Venn diagrams showing the extent of overlap between different combinations of search engines. a) and b) represent overlap of unique identified peptide sequences and PSMs, respectively. Note that iProphet filters out low-scoring PSMs and peptides. [file 12859_2014_441_MOESM1_ESM.docx]

**SUPPLEMENTARY MATERIAL**

**APP: an Automated Proteomics Pipeline for the analysis of mass spectrometry data based on multiple open access tools**

by

**Erik K Malm, Vaibhav Srivastava, Gustav Sundqvist and Vincent Bulone***

## Address for all co-authors:

## *Division of Glycoscience, School of Biotechnology, Royal Institute of Technology (KTH), AlbaNova University Centre, Stockholm, Sweden.*

## ^*^For correspondence. E-mail: bulone@kth.se; Tel. (+46) 8 5537 8841; Fax (+46) 8 5537 8468.

**E-mail of all co-authors:**

Erik K Malm: emalm@kth.se;

Vaibhav Srivastava: vaibhav.srivastava@biotech.kth.se

Gustav Sundqvist: gustavs@kth.se

Vincent Bulone: bulone@kth.se

| **Software name** | **Version** | **Reference** |
| --- | --- | --- |
| X!Tandem | X! TANDEM CYCLONE TPP (2011.12.01.1) | [[1](#XCraig2004)] |
| Myrimatch | MyriMatch 2.1.138 | [[2](#XTabb2007)] |
| Comet | Comet version ”2013.02 rev. 0” | [[4](#XEng2013)] |
| MS-GF+ | v1.0 (v8299) | [[7](#XKim2010)] |
| SpectraST | V 4.0, TPP v4.6 OCCUPY rev 3 | [[6](#XLam2007)] |
| TPP base (PeptideProphet, ProteinProphet, iProphet) | TPP v4.6 OCCUPY rev 3 | [16] |

**Table S1 Software versions used for example tasks.**

The search engine output shows clearly that each search engine matches a subset of unique spectra, often corresponding to a set of unique peptides (Fig. S1a). For example, MS-GF+ matches 883 peptides not found by any other search engine (Fig. S1a). Other search engines have various degrees of overlap, but all match a significant amount of unique stripped peptide sequences. When looking at the PSM level, the differences are even more pronounced, with only 1278 PSMs identified by all search engines out of over 13000 in total (Fig. S1b). It should be noted that results are dependent on the data and settings used. Because of this, any result should be seen as a single case study rather than a generalization of abilities.


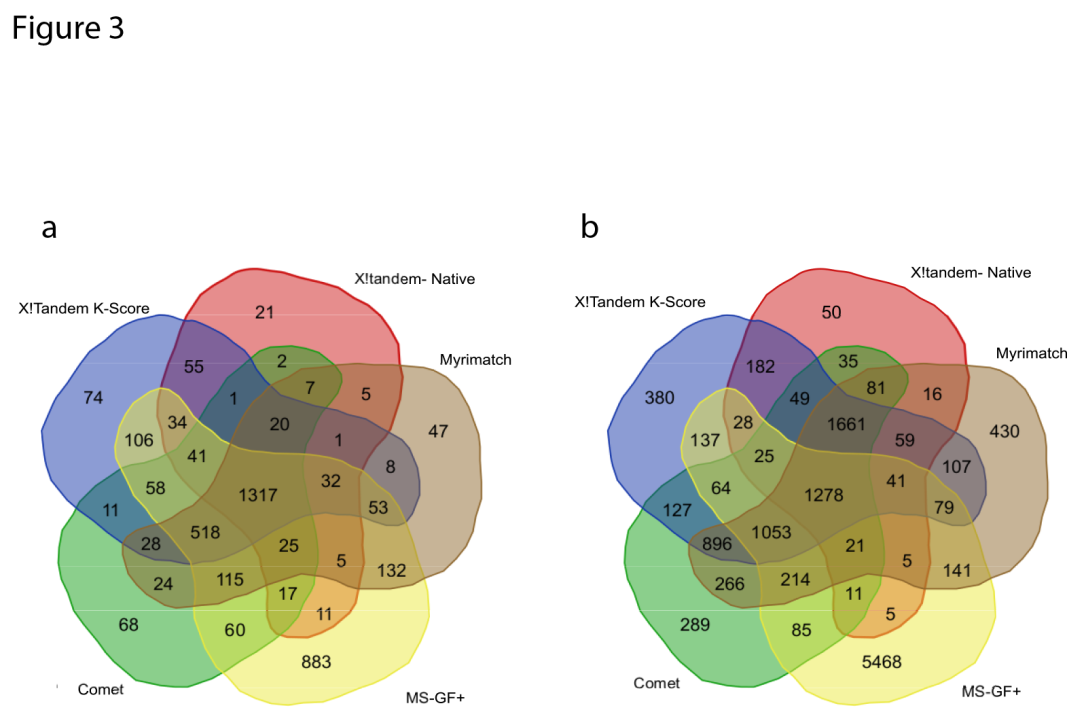


**Figure S1** **Output of the example workflow.** a) Number of identified peptide sequences by each search engine; while many peptides are common, each search engine provides a unique set of identified peptides. b) Equivalent output as in a), but for PSMs; the figure highlights matched spectra rather than peptide sequences.

The best search engine in terms of PSM and peptide matches was MS-GF+, as judged by comparison with the iProphet combined output of all search engines plus Spectral Search (Fig. S2). Combining search engine output boosted the number of identified PSMs passing the quality threshold from 8655 for MS-GF+ to 13029 (Table 1, main text). In this case, the inclusion of spectral search modestly boosted the PSM number to 13232, matching 3501 unique identified peptides (Table 1, main text).


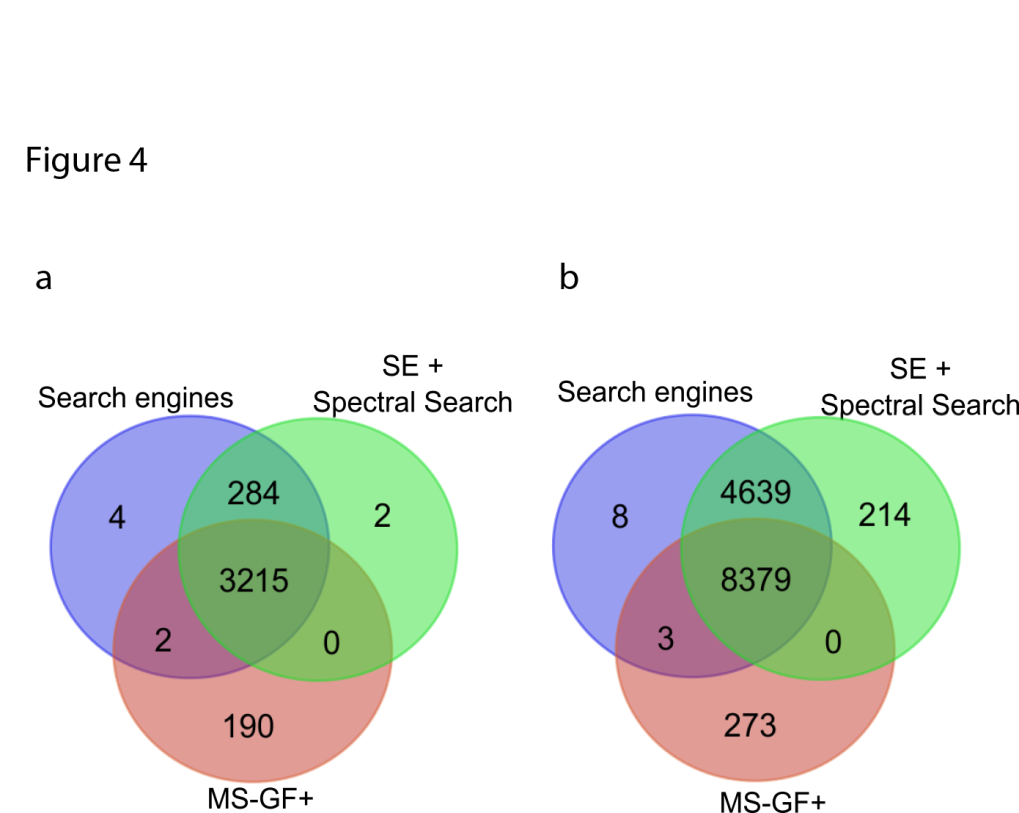


**Figure S2 Output of the combined workflow.** Venn diagrams showing the extent of overlap between different combinations of search engines. a) and b) represent overlap of unique identified peptide sequences and PSMs, respectively. Note that iProphet filters out low-scoring PSMs and peptides.
